# Fungal Chitin Reduces Platelet Activation Mediated via TLR8 Stimulation

**DOI:** 10.3389/fcimb.2019.00383

**Published:** 2019-11-12

**Authors:** Jordan Leroy, Clovis Bortolus, Karine Lecointe, Melissa Parny, Rogatien Charlet, Boualem Sendid, Samir Jawhara

**Affiliations:** ^1^INSERM, U995/Team2, Lille, France; ^2^Lille Inflammation Research International Centre, University of Lille, U995-LIRIC, Lille, France; ^3^Service de Parasitologie Mycologie, Pôle de Biologie Pathologie Génétique, CHU Lille, Lille, France

**Keywords:** *Candida albicans*, chitin, platelets, neutrophils, Toll-like receptor, P-selectin, thrombin, aggregation

## Abstract

Platelets play an important role in the innate immune response. During candidaemia, circulating fungal polysaccharides, including chitin, are released into the bloodstream and can interact with platelets and induce modulation of platelet activities. However, the role of circulating chitin in platelet modulation has not been investigated. The aims of the present study were to assess the effect of fungal chitin on activation, adhesion, aggregation and receptor expression of platelets and their impact on the host defense against *Candida albicans*. Platelets pre-treated with different concentrations of chitin (10–400 μg/mL) extracted from *C. albicans* were analyzed in terms of activation, Toll-like receptor (TLR) expression, aggregation and adhesion to *C. albicans*. Chitin treatment reduced platelet adhesion to *C. albicans* and neutrophils. P-selectin expression was significantly decreased in platelets challenged with chitin. Aggregation and intracellular Ca^2+^ influx were also decreased in platelets. TLR8 mRNA and proteins were expressed in platelets pre-treated with chitin when compared to untreated platelets. Overall, chitin purified from *C. albicans* reduced the adhesion, activation and aggregation of platelets mediated via TLR8 stimulation by decreasing intracellular Ca^2+^ influx and P-selectin expression.

## Introduction

Platelets play a crucial role in haemostasis and wound healing (Livio et al., 1988). They are activated by multiple extracellular signals. On an injured endothelial surface, platelets adhere to the exposed subendothelial matrix through a large repertoire of adhesion receptors that lead to platelet activation, resulting in conformational changes to integrin α_IIb_β_3_ that mediate platelet aggregation by binding to soluble fibrinogen. During this process, platelets start to change their shape by the formation of pseudopods when intracellular Ca^2+^ concentration exceeds a specific threshold and release the contents of their granules including P-selectin (Livio et al., 1988; Guidetti et al., 2019).

Platelets also participate in different immunological processes, including intervention against microbial threat. Platelets interact with various pathogenic fungi including *Candida* species. It has been reported that intravenous (IV) injection of different *Candida* species in mice resulted in the rapid adhesion of platelets to *Candida* and the generation of *C. albicans* pseudohyphal forms (Robert et al., 2000).

In the rabbit model, IV injection of *Candida* results in the formation of massive vegetations and promotes the adhesion of *C. albicans* to fibrin-platelet-erythrocyte deposits (Calderone et al., 1978). *C. albicans* is a human commensal yeast and a natural saprophyte of the digestive and vaginal microbiota. Excessive colonization of the digestive mucosa by *C. albicans* is associated with multiple risk factors including immunosuppression and alteration of the mucosal barrier promoting translocation of the yeast through the digestive tract into the blood leading to severe invasive fungal infections. Interaction between the fungus and platelets involves the cell wall, which consists predominantly of polysaccharides associated with proteins and lipids (Gow et al., 2012). Its innermost layers are formed from a dense network of polysaccharides consisting of chitin and β-glucans, which are responsible for the resistance of the cell wall to chemical agents and mechanical action (Gow et al., 2012).

During infection, fungal cell wall components are released into the bloodstream and can be detected up to 10 days before the onset of clinical signs of invasive candidiasis (Sendid et al., 2008, 2013). The fungal cell wall is a dynamic structure and is in perpetual change (Poulain et al., 2009). During hyphal formation, *C. albicans* contains 3–4 times more chitin than in its yeast phase.

Chitin is the second most abundant natural biopolymer of β-1,4-N-acetylglucosamine (GlcNAc) in the world (Park and Kim, 2010). This polysaccharide is an essential component of the exoskeleton of arthropods and crustaceans and the fungal cell wall including that of *C. albicans*, contributing to its rigidity and viability (Mora-Montes et al., 2011). Oligomers of chitin or chito-oligosaccharides exert antimicrobial, anti-tumor and immunomodulatory properties (Park and Kim, 2010). Activation of dectin-1 and Toll-like receptor 2 (TLR2) in macrophages by *C. albicans* results in the activation of a signaling cascade that leads to chitotriosidase secretion that degrades chitin into small fragments. These chitins are recognized by NOD-2 and TLR9 receptors leading to the production of the anti-inflammatory cytokine interleukin (IL)-10 (Wagener et al., 2014). TLRs play a fundamental role in recognizing fungal cell wall components. TLR1, -2, and -4 have been widely described to recognize *C. albicans* (Netea et al., 2004; Choteau et al., 2017). Vancraeyneste et al. showed that soluble short fractions of β-glucans derived from *C. albicans* inhibit platelet activation mediated by TLR4 (Vancraeyneste et al., 2016). Clinically, chitins are released into the blood during fungal infection. Anti-chitin antibodies have also been detected in the serum of patients with candidaemia (Sendid et al., 2008; Poulain, 2015), but the role of chitin in platelet modulation has not yet been investigated (Sendid et al., 2008, 2013). In the current study, we investigated the effects of *C. albicans* chitin on platelet modulation in terms of adhesion, aggregation, activation and receptor expression.

## Materials and Methods

### Ethics Statement

Healthy donors were informed and gave their written consent to participate in the present study. The study protocol was reviewed and approved by the Ethics Committee of Lille University Hospital. The study was conducted according to the principles expressed in the Declaration of Helsinki.

### Preparation of Washed Platelets

Whole blood was collected from healthy donors. Platelets were isolated by differential centrifugation and then washed in modified Tyrode's buffer (2.7 mM KCl, 3.3 mM Na_2_HPO_4_, 137 mM NaCl, 1.2 mM NaHCO_3_, 1 mg/ml bovine serum albumin, 3.8 mM HEPES, 5 mM glucose) (Byzova and Plow, 1997).

### *C. albicans* Strain and Culture Conditions

*C. albicans* strain SC5314 was used in this study and maintained at 4°C in yeast peptone dextrose broth (YPD; 1% yeast extract, 2% peptone, 2% dextrose). To prepare the yeast suspension, *C. albicans* cells were cultured in Sabouraud dextrose broth (Sigma-Aldrich, St. Quentin Fallavier, France) for 24 h at 37°C in a rotary shaker. *C. albicans* cells were harvested by centrifugation. After washing several times with phosphate-buffered saline (PBS), *C. albicans* cells were resuspended in PBS.

### Preparation of Chitin From *C. albicans*

*C. albicans* cell pellets were washed twice in PBS. Chitin was extracted from *C. albicans* yeast cells as described previously (Charlet et al., 2018b). Briefly, 20 mL of 10% KOH was added to the *C. albicans* cell pellet. This procedure was repeated twice and the pellet was autoclaved at 120°C for 2 h. After washing with distilled water, the supernatant was removed and 50% acetic acid and 50% hydrogen peroxide were added to the pellet, which was then autoclaved at 120°C for 2 h. After washing with distilled water, the pellet was centrifuged and the chitin fraction was lyophilized. Nuclear magnetic resonance (NMR) analysis was performed to confirm the nature of the chitin. Intact chitin purified from *C. albicans* was added to deuterated hexafluoroisopropanol (Euriso-Top) at 70°C until dissolved. All experiments were performed using a Bruker Avance 600 MHz (13.1 T) spectrometer with Bruker standard pulse programs. MALDI-TOF mass spectra were acquired on a Voyager Elite DE-STR mass spectrometer (Perspective Biosystems, Framingham, MA). A mixture of matrix (1 μL containing 10 mg/mL DHB and 5% acetonitrile) and 1 μL of sample was deposited on a MALDI plate. Small and large soluble chitin fragments were detected by MALDI-TOF mass spectrometry. A BiCinchoninic acid assay was employed to determine the chitin concentration extracted from *C. albicans* (Le Devedec et al., 2008). The standard range for the N-acetyl-glucosamine (GlcNAc) control was 0.1–5 mg/mL.

### Platelet Adhesion to Neutrophils or *C. albicans*

Neutrophils were prepared from the peripheral blood of healthy donors by gradient centrifugation, according to the protocol of Pluskota et al. (2003). Fresh platelet poor plasma (200 μL) was added to each well of a 96-well plate and incubated for 2 h at 37°C (Clark et al., 2007). After several washes, a suspension of 10^6^ neutrophils in 200 μL of RPMI medium containing 1 mM Mg^2+^/Ca^2+^ was added to each well of a 96-well plate coated with platelets. Non-adherent cells were removed and the wells were washed with Hank's balanced salt solution (HBSS) (Bouaouina et al., 2004). To assess platelet adhesion to *C. albicans*, 200 μL of RPMI medium containing 10^5^ *C. albicans* cells was added to each well of a 96-well plate. This plate was then incubated at 37°C to allow *C. albicans* to adhere to the bottom of the plate. Thrombin-stimulated platelets (10^6^ cells/mL) were labeled with calcein and then pre-treated for 30 min with chitin (20–200 μmol/L) incubated at 37°C for 20 min (Clark et al., 2007). After several washes, the percentage platelet adhesion to *C. albicans* or neutrophils was examined using a fluorometer (FLUOstar® Omega; BMG Labtech, Champigny sur Marne, France). To examine platelet adhesion to *C. albicans* by confocal microscopy, washed human platelets were stained with calcein and *C. albicans* cells were labeled with 5B2 monoclonal antibody (1:2,000 dilution) (Vancraeyneste et al., 2016). Slides were examined by confocal microscopy (Zeiss LSM710). For THP-1 cell culture, THP-1 cells were incubated in RPMI 1640 medium (Gibco by Life Technologies™, France) supplemented with 10% fetal bovine serum, 50 IU/mL penicillin and 50 IU/mL streptomycin. THP-1 cells were differentiated into macrophages in the presence of phorbol-12-myristate 13-acetate (100 nM; Sigma-Aldrich, St. Quentin Fallavier, France) at 37°C and 5% CO_2_.

### Platelet Aggregation Assay

Washed human platelets (10^8^ cells/mL) were pre-treated with chitin (20 μmol/L) for 30 min in HBSS and then treated with thrombin (0.2 U/mL; Sigma-Aldrich, St. Quentin Fallavier, France) to promote platelet aggregation (Cambi et al., 2008). Platelet aggregation was assessed continuously after thrombin addition for 5–10 min using an aggregometer (Labor Biomedical Technologies Gmbh, LABITEC APACT 4004).

### Intracellular Ca^2+^ Measurements

Ca^2+^ measurements were performed as described previously (Hussain and Mahaut-Smith, 1999). Briefly, 5 μM fura-2 acetoxymethyl ester (Invitrogen, France) and 0.2 μg/mL Pluronic F-127 were added to washed platelets pre-treated with different concentrations of chitin (20, 50, 100, or 200 μg/mL) in HBSS without Ca^2+^ at 37°C for 30 min. Platelets were then washed once and resuspended in HBSS. After platelet activation with thrombin (0.05 U/mL), the Ca^2+^ response was measured using a fluorometer (FLUOstar® Omega; BMG Labtech, Champigny sur Marne, France). The ratio of absorbance at 340/380 nm was converted into Ca^2+^ concentration (Ca^2+^).

### Analysis of the Expression/Activation of Platelet Receptors and Cell Wall Surface Glycan Expression of *C. albicans*

This was performed in RPMI medium (300 μL) containing 5 × 10^5^ platelets/mL; RPMI medium does not induce platelet activation (Speth et al., 2013). Platelets were pre-treated with chitin at different concentrations (20–200 μmol/L) for 2 min and then activated with thrombin (0.05 U/mL). The activation or expression of platelet receptors was assessed using specific or isotype antibodies linked to fluorochromes (anti-human/mouse CD62P P-selectin, anti-human PAC-1, anti-mouse PE IgG1k, Brilliant Violet 421 anti-human CD41; Ozyme, France). After incubation for 30 min in the dark, platelets were washed twice with 300 μl PBS. The platelets were resuspended in 300 μL PBS. Specific isotype controls (mouse IgG1, κ Isotype Control Biolegend®, and mouse IgG1, κ Isotype Control BD Parmagen™) were used in each flow cytometry experiment. The expression/activation of platelet receptors was determined by flow cytometry (BD LSRFortesa® X20). Unstimulated or stimulated platelets with thrombin (0.05 U/mL) were used as controls for each experiment. The data obtained were analyzed using Kaluza software®. To examine the expression of platelet receptors by confocal microscopy, washed human platelets were labeled with TLR monoclonal antibodies (Vancraeyneste et al., 2016). Specific slides (wells 6.7 mm; Thermo Scientific, France) were used in this experiment and the coverslips were examined by confocal microscopy (Zeiss LSM710, Zeiss Airyscan SR mode x6311.4).

### Real-Time PCR and Western Blot

A Nucleospin RNA/Protein kit (Macherey-Nagel, France) was used to isolate total RNA and proteins from platelets. Quantification of RNA in each sample was performed by spectrophotometry (Nanodrop; Nyxor Biotech, France). Reverse transcription of mRNA was performed in a final volume of 20 μL containing 1 μg total RNA (high capacity cDNA RT kit; Applied Biosystems). cDNA synthesis was performed according to the High Capacity DNA Reverse Transcription (RT) protocol, using Master Mix (Applied Biosystems). SYBR green dye intensity was determined using one-step software (Jawhara et al., 2008). All results were normalized to the reference gene, *GAPDH*. For Western blot analysis, proteins were recovered in lysis buffer (Tris/HCl 50 mM; Triton-X100, 1%; SDS 0.1%; EDTA 5 mM; NaCl 150 mM). The protein content of each sample was estimated using a BiCinchoninic acid protein assay (Pierce) and adjusted to the same protein concentration prior to analysis by SDS-PAGE on a 10% acrylamide gel [18]. Proteins are transfered onto a nitrocellulose membrane (iBlot 2; Thermofisher Scientific). Membranes were blocked with TBS-BSA 3% for 1 h and then incubated overnight with monoclonal anti-TLR (dilution 1:250) (TLR4 Biotechne® TLR8 GeneTex®). Horseradish peroxidase-labeled secondary antibody (1:5,000 dilution) (Southern Biotech) were used to detect monoclonal antibodies.

### Statistical Analysis

Statistical analysis was performed using XLSTAT and Prism 4.0 (GraphPad). Data were analyzed using the Mann-Whitney *U* test to compare pairs of groups. The results are expressed as the mean ± SD of individual experimental groups. Differences were considered significant when the *P*-value was < 0.05.

## Results

### Effect of Fungal Chitin on Platelet-*C. albicans* or Platelet-Neutrophil Interactions

Platelets can bind directly to different pathogens through different surface glycoproteins. In order to evaluate the effect of chitin on the *C. albicans*-platelet adhesion process, we used an increasing concentration of fungal chitin ranging from 10 to 200 μg/mL.

These different chitin concentrations were used in the present study as there as there is no routine clinical test to measure the concentration of circulating chitin in the blood of patients with candidaemia.

**Figure 1:**
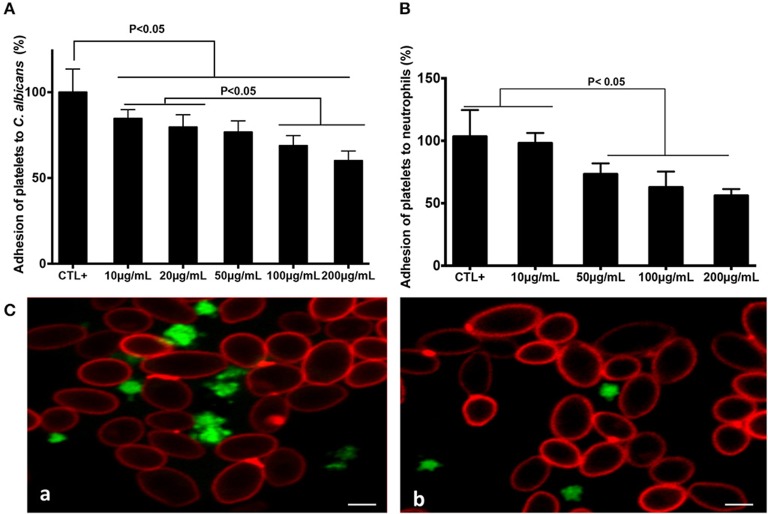
Chitin reduced platelet adhesion to *C. albicans* and neutrophils. Platelet adhesion to *C. albicans* **(A)** or to neutrophils **(B)**. Calcein-labeled washed platelets (10^6^ cells/mL) stimulated with thrombin (0.05 U/mL), and untreated as the control (CTL+) or pre-treated with chitin at different concentrations (10, 20, 50, 100, or 200 μg/mL), were incubated with either *C. albicans* or neutrophils. The data obtained from the adhesion of thrombin-activated platelets (CTL+) to *C. albicans* or to neutrophils were assigned a value of 100%. Data are the mean ± SD of five independent experiments (*n* = 3–4 per group). **(C)** Representative images of platelet adhesion to *C. albicans*. Calcein-labeled washed platelets (green) were untreated with chitin, but received only HBSS (a), or were pre-treated with chitin (b) and then incubated with *C. albicans*. *C. albicans* was stained with anti-phycoerythrin *C. albicans* antibody (5B2). Scale bar = 5 μm.

Increasing concentrations of fungal chitin were associated with a decrease in percentage platelet adhesion to *C. albicans* (Figure 1). This decrease was significant for chitin concentrations ≥20 μg/mL. Activated platelets are crucial for the activation of neutrophils and in neutrophil-mediated inflammatory responses. The impact of chitin on platelet-neutrophil interactions was assessed. Platelets pre-treated with chitin (50, 100, or 200 μg/mL) showed a significant decrease in adhesion to neutrophils (Figure 1).

### Effect of Chitin on Platelet Aggregation

**Figure 2:**
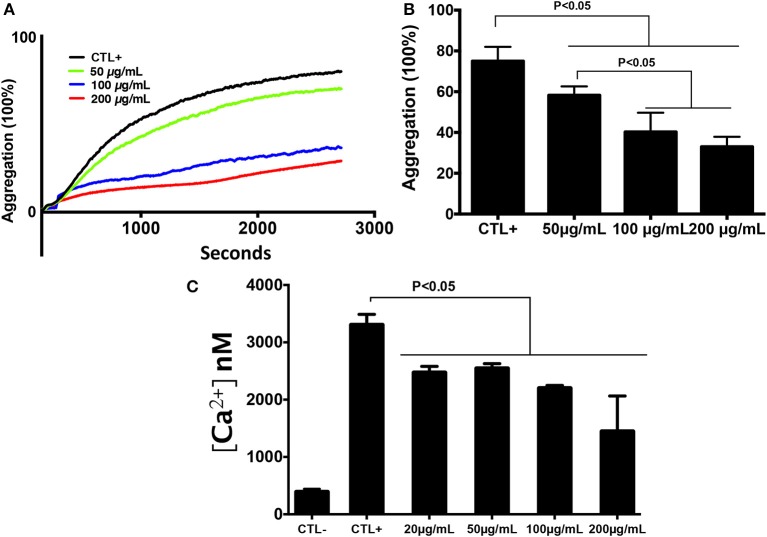
Chitin reduced platelet aggregation and intracellular calcium concentration. **(A)** Representative aggregation curve. Untreated platelets or platelets pre-treated with chitin at different concentrations (50, 100, or 200 μg/mL) were activated with 0.2 U/mL thrombin. **(B)** Percent maximal aggregation in the presence of thrombin. Data are the mean ± SD of three independent experiments (*n* = 3–4 per group). **(C)** Fura-2 fluorescence reflecting cytosolic Ca^2+^ concentration [Ca^2+^] of platelets pre-treated with chitin and stimulated with thrombin.

In order to determine the effect of chitin on platelet aggregation involving α_IIb_β_3_ integrin, platelets were pre-exposed to different chitin concentrations (50, 100, or 200 μg/mL) for 30 min and then stimulated with 0.2 U/mL thrombin (Figure 2A). A decrease in the percentage of platelet aggregation was observed in the presence of fungal chitin with a dose-dependent effect. There was a significant decrease in aggregation in the presence of chitin at a concentration of 100 or 200 μg/mL (Figure 2B).

### Effect of Chitin on Platelet Activation and Receptor Expression

**Figure 3:**
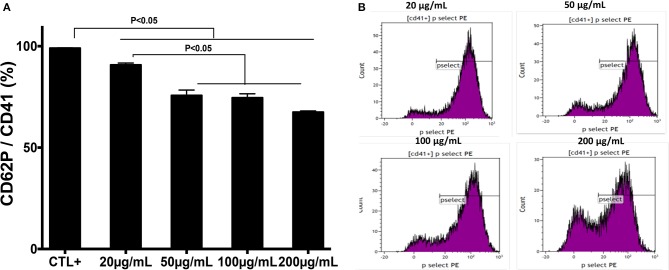
Pre-treatment of platelets with chitin decreases P-selectin expression. **(A)** Platelets (5 × 10^5^ cells/mL) were activated with thrombin (0.05 U/mL) and pre-treated with chitin at a concentration of 20, 50, 100, or 200 μg/mL. CTL+ corresponds to thrombin-activated platelets without chitin treatment. **(B)** The red curve shows the thrombin-activated platelet population treated with chitin at 20, 50, 100, or 200 μg/mL and detected with anti-P-selectin antibodies. Curves are representative of three separate experiments.

The effect of chitin on platelet activation was determined using fura-2, which exhibits an increase in fluorescence intensity on binding to intracellular Ca^2+^. The concentration of intracellular calcium in platelets stimulated with thrombin (0.05 U/mL) was higher than that in unstimulated platelets (Figure 2C). Pre-treatment of platelets with chitin decreased the concentration of intracellular Ca^2+^ (Figure 2C). P-selectin expression, which is a crucial marker of platelet activation, was also investigated. Platelets were labeled with a specific CD41 marker by targeting the 2b chain of the alpha integrin of the GPIIb-IIIa complex. In the presence of thrombin, the majority of the CD41^+^ platelet population expressed P-selectin while platelets pre-treated with chitin showed a significant decrease in platelet activation proportional to the fungal chitin concentration, with a greater effect of chitin at a concentration of 200 μg/mL suggesting that chitin has a dose-dependent effect (Figure 3). In parallel, the activation of integrin α_IIb_β_3_ was assessed in thrombin-stimulated platelets pretreated with chitin at a concentration of 20 μg/mL (Supplementary Data). A significant decrease in integrin α_IIb_β_3_ activation was observed in thrombin-stimulated platelets when compared to those untreated with chitin. These data corroborate the aggregation assay showing that chitin pre-treatment reduces platelet aggregation.

**Figure 4:**
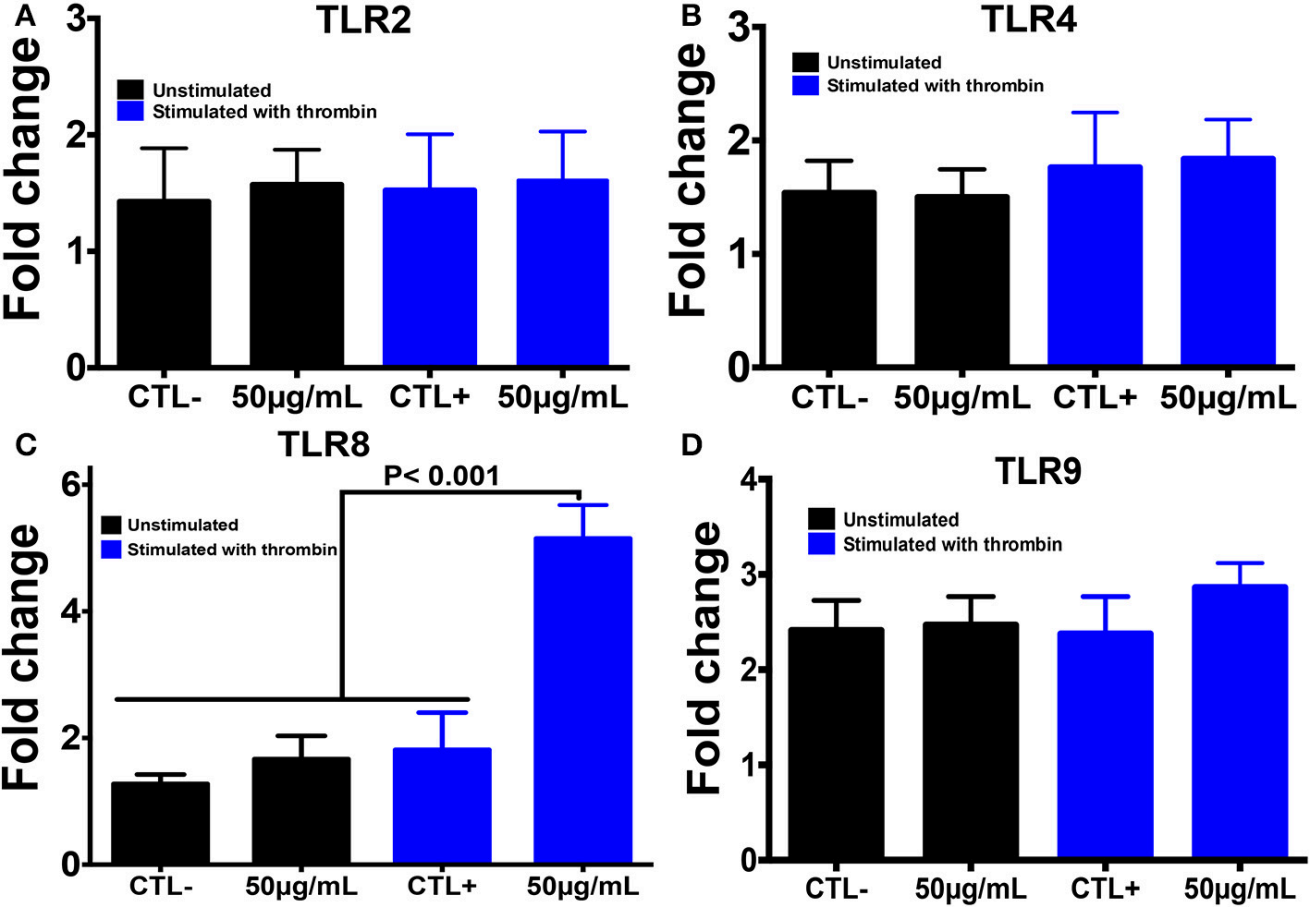
Chitin increases TLR8 expression in thrombin-stimulated platelets. **(A–D)** Relative expression levels of TLR2, TLR4, TLR8, and TLR9 mRNA, respectively, in platelets by real-time PCR. CTL– (control) corresponds to unstimulated platelets, CTL+ (control) represents platelets stimulated with thrombin (0.05 U/mL). 50 μg/mL corresponds to chitin concentration. Data are the mean ± SD of three independent experiments.

Using PCR, we also investigated the expression of different TLRs that are known to sense many PAMP molecules, including chitin, promoting the host defense against pathogens (Figure 4). Western blot was then performed to confirm the expression of these receptors. A histone marker was used to exclude the presence of leukocytes in the platelet isolate. The human macrophage-differentiated THP-1 cell line was employed as a control. Platelet isolation was histone-free, indicating that the platelet preparation was not contaminated with leukocytes. In terms of mRNA expression levels for TLR2, TLR4, and TLR9, no significant difference was observed between platelets pre-treated with chitin (50 μg/mL) and untreated platelets (Figure 4). In contrast, TLR8 mRNA was highly expressed in thrombin-activated platelets pre-treated with chitin compared to that in untreated platelets, and this expression was concentration-dependent, indicating that chitin and thrombin stimulation modulate TLR8 mRNA expression.

**Figure 5:**
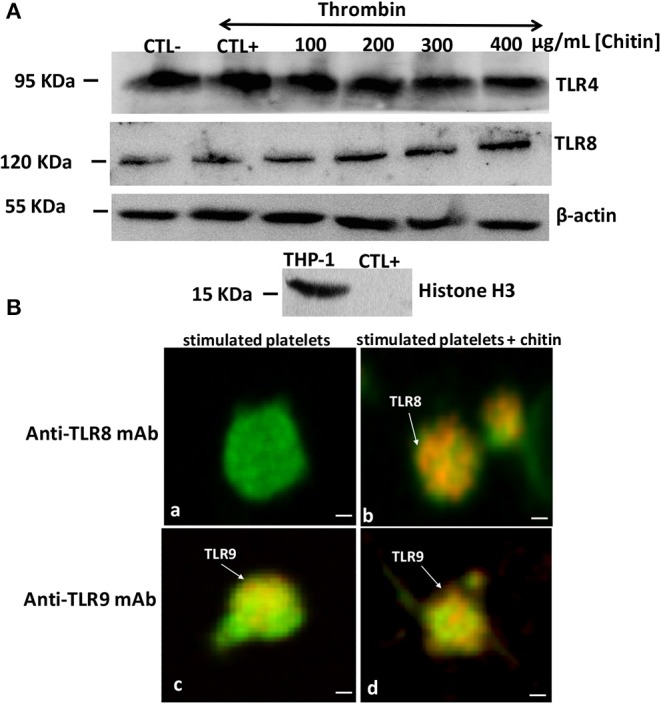
Detection of TLR4 and TLR8 proteins in platelets by Western blot. **(A)** Platelets (10^6^ cells/mL) without chitin treatment or thrombin stimulation (CTL–), platelets activated with thrombin at a concentration of 0.05 U/mL (CTL+), platelets activated with thrombin and pre-treated with chitin at different concentrations (100, 200, 300, or 400 μg/mL). Human macrophage-differentiated THP-1 cells used as a control and platelets were labeled with histone marker to exclude the presence of leukocytes in the platelet isolate. Western blots are representative of three independent experiments. **(B)** Representative images of TLR8 detection in platelets. (a,c) Calcein-labeled thrombin-stimulated platelets (green), untreated with chitin and labeled with either anti-TLR8 or anti-TLR9 antibody (red). (b,d) Calcein-labeled thrombin-stimulated platelets (green), pre-treated with chitin and labeled with either anti-TLR8 or anti-TLR9 antibody (red). Scale bar = 1 μm.

The mRNA data correlated with those of Western blot analysis showing that TLR4 expression is independent of chitin exposure while high expression levels of TLR8 were detected in thrombin-activated platelets pre-treated with increasing concentrations of chitin (Figure 5). We performed quantification of Western Blot band intensity corresponding to platelets alone (CTL–), platelets in the presence of thrombin (CTL+) and thrombin-stimulated platelets pre-treated with chitin at different concentrations, using ImageJ bundled with 64-bit Java 1.8.0-112. The intensity of each band was normalized to that of beta-actin. We observed an increase in TLR8 band intensity with an increase in chitin concentration (Supplementary Data). In parallel, we examined the expression of TLR8 and TLR9 platelet receptors by confocal microscopy. In contrast to TLR9, TLR8 expression was only detected in thrombin-activated platelets pre-treated with chitin (Figure 5B).

## Discussion

Circulating fungal polysaccharides are in contact with immune cells, including platelets, and these polysaccharides play an important role in modulation of the host response (Poulain et al., 2009; Poulain, 2015). Different studies show that β-glucans derived from *C. albicans* play a crucial role in modulation of the host response, including platelets, but little is known about the effect of fungal chitin on platelet modulation (Jawhara et al., 2012; Vancraeyneste et al., 2016; Charlet et al., 2018a). In the present study, we assessed the effect of chitin purified from the cell wall of *C. albicans* on platelet modulation. During infection, platelets interact directly with leukocytes or pathogens. Pre-treatment of platelets with fungal chitin decreased platelet adhesion to *C. albicans* but also to neutrophils, suggesting that chitin promotes the escape of *C. albicans* from immune cells. We also showed that after platelet activation by thrombin, intracellular Ca^2+^ influx increased in platelets. This influx led to platelet aggregation while pre-treatment of platelets with chitin reduced intracellular Ca^2+^ and platelet aggregation. Different studies show that platelet aggregation and enclosure of microorganisms in platelet-fibrin matrices offer protection to microorganisms from antibiotics or clearance by leukocytes (Clawson and White, 1971; Maisch and Calderone, 1981). Maish et al. showed that mannan derived from the *C. albicans* cell wall is involved in the adherence of *C. albicans* to fibrin-platelet matrices, which form *in vivo* on the endocardium of heart valves (Maisch and Calderone, 1981). It has been reported that β-1,3 glucans purified from *C. albicans* reduced platelet aggregation and platelet-*C. albicans* and platelet-neutrophil interactions, which protected *C. albicans* from leukocyte activation (Vancraeyneste et al., 2016).

P-selectin expression promotes the adhesion of platelets to leukocytes (Duerschmied et al., 2013; Kral et al., 2016). This platelet-leukocyte interaction induces the recruitment of neutrophils in infected tissues and increases the microbicidal activity of neutrophils (Kral et al., 2016). In this study, a decrease in P-selectin expression was observed in platelets pre-treated with chitin. These data corroborate those for aggregation and platelet adhesion to neutrophils, indicating the involvement of chitin in inhibiting platelet activation.

It is known that fungal polysaccharides can modulate the inflammatory response through their interactions with TLRs, but it remains unclear how fungal chitin modulates TLR expression in platelets (Choteau et al., 2017; Fuchs et al., 2018). TLRs play an important role in the recognition of PAMPs, including fungal chitin, promoting platelets to recognize pathogens in addition to their role in haemostasis (Ward et al., 2005; Schattner, 2019). Activation of platelets with thrombin induces an important modulation of TLR1, TLR6, and TLR9 during vascular lesions potentially mediated by bacterial infection (Shiraki et al., 2004). It has been shown that chitin induces TLR4 expression on keratinocytes at the mRNA and protein level (Koller et al., 2011). Wagener et al. demonstrated that chitin derived from *C. albicans* has the potential to attenuate the inflammatory response via TLR-9 and NOD-2 signaling in macrophages (Wagener et al., 2014). Additionally, Mora-Montes et al. showed that PBMC-treated fungal chitin blocked normal recognition of *C. albicans* cells (Mora-Montes et al., 2011). In a dextran sulfate sodium-induced colitis model, oral administration of fungal chitin reduced the inflammatory parameters and leukocyte infiltration into the gut mucosa and increased IL-10 production via stimulation of NOD-2 and TLR8 (Vancraeyneste et al., 2016). In the present study, a large panel of TLRs receptors were analyzed in platelets by RT-q-PCR (Supplementary Data). In contrast to macrophages differentiated from Thp1 cells pre-treated with chitin, which showed high mRNA transcript and protein expression of TLR2 (data not shown), we did not observe any increase in TLR2 mRNA transcripts or protein expression in thrombin-activated platelets pre-treated with chitin. TLR4 was expressed independently of chitin treatment. Of note, treatment of inactivated platelets with chitin did not show any significant increase in TLR8 mRNA levels, while TLR8 transcript levels were significantly increased in thrombin-activated platelets pre-treated with chitin. Additionally, TLR8 mRNA transcript levels in activated platelets were correlated with protein expression levels of TLR8 in a chitin concentration-dependent manner. These data suggest that fungal chitin promotes an increase in TLR8 in thrombin-activated platelets.

In conclusion, chitin purified from *C. albicans* reduces the adhesion, activation and aggregation of activated platelets mediated via TLR8 stimulation by decreasing intracellular Ca^2+^ influx and P-selectin expression. Overall, this study offers a new insight into the role of chitin in modulating platelet activities and platelet-neutrophil interactions, promoting the escape of *C. albicans* from the host defense.

## Data Availability Statement

All datasets generated for this study are included in the article/Supplementary Material.

## Ethics Statement

The studies involving human participants were reviewed and approved by the Ethics Committee of Lille University Hospital. The patients/participants provided their written informed consent to participate in this study.

## Author Contributions

JL, CB, KL, MP, RC, and SJ performed the experiments. JL, CB, KL, MP, RC, BS, and SJ analyzed the data and interpreted the results of the experiments. SJ designed the experiments and drafted the manuscript.

### Conflict of Interest

The authors declare that the research was conducted in the absence of any commercial or financial relationships that could be construed as a potential conflict of interest.

## Abbreviations

*C. albicans*: *Candida albicans*
CTL: control
DNA: deoxyribonucleic acid
mAb: monoclonal antibody
PAMPs: pathogen associated molecular patterns
PBS: phosphate-buffered saline
RNA: ribonucleic acid
TLR: Toll-like receptor.
