# Supplementary material for: Fungal Chitin Reduces Platelet Activation Mediated via TLR8 Stimulation
Source: Front Cell Infect Microbiol. 2019 Nov 12;9:383. doi: 10.3389/fcimb.2019.00383 (PMC6861417; doi:10.3389/fcimb.2019.00383)
Supplement: Supplementary file 2 [file Data_Sheet_2.docx]

**Fungal chitin reduces platelet activation mediated via TLR8 stimulation**

Jordan Leroy, Clovis Bortolus, Karine Lecointe, Melissa Parny , Rogatien Charlet, Boualem Sendid, Samir Jawhara

**Supplementary data**

**
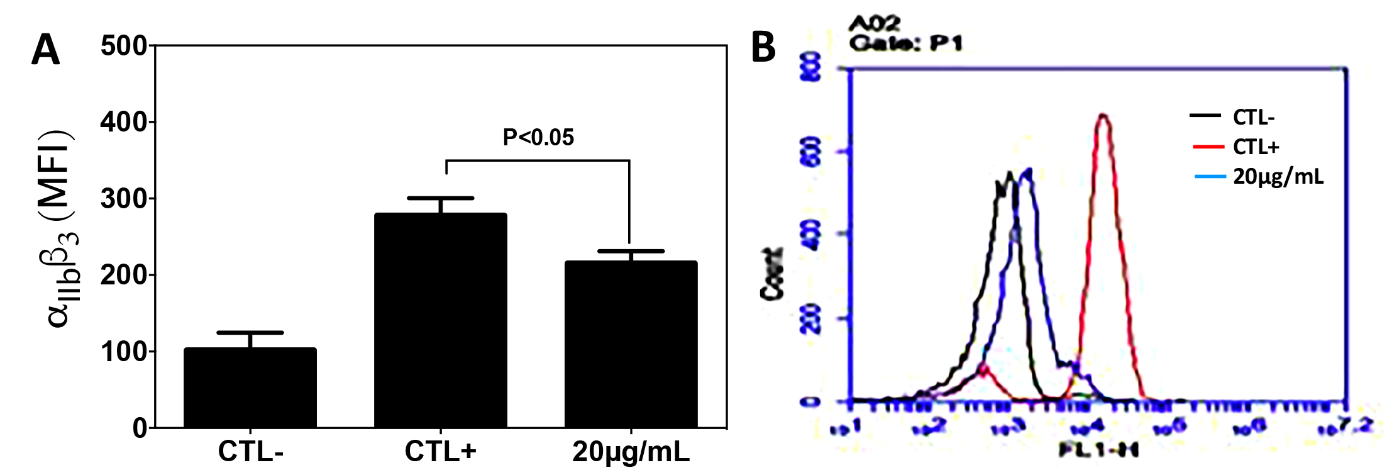
**

**Figure 1: Chitin showing a decrease in α_IIb_β_3_ integrin activation. (A and B)** Platelets (5 x 10^5^ cells/mL) were activated with thrombin (0.05 U/ml) and pretreated with chitin at a concentration of 20 µg/mL. CTL- (control) corresponds to unstimulated platelets. CTL+ (control) corresponds to thrombin-activated platelets without chitin pretreatment. 20 µg/mL shows the thrombin-activated platelet population pretreated with chitin.

**
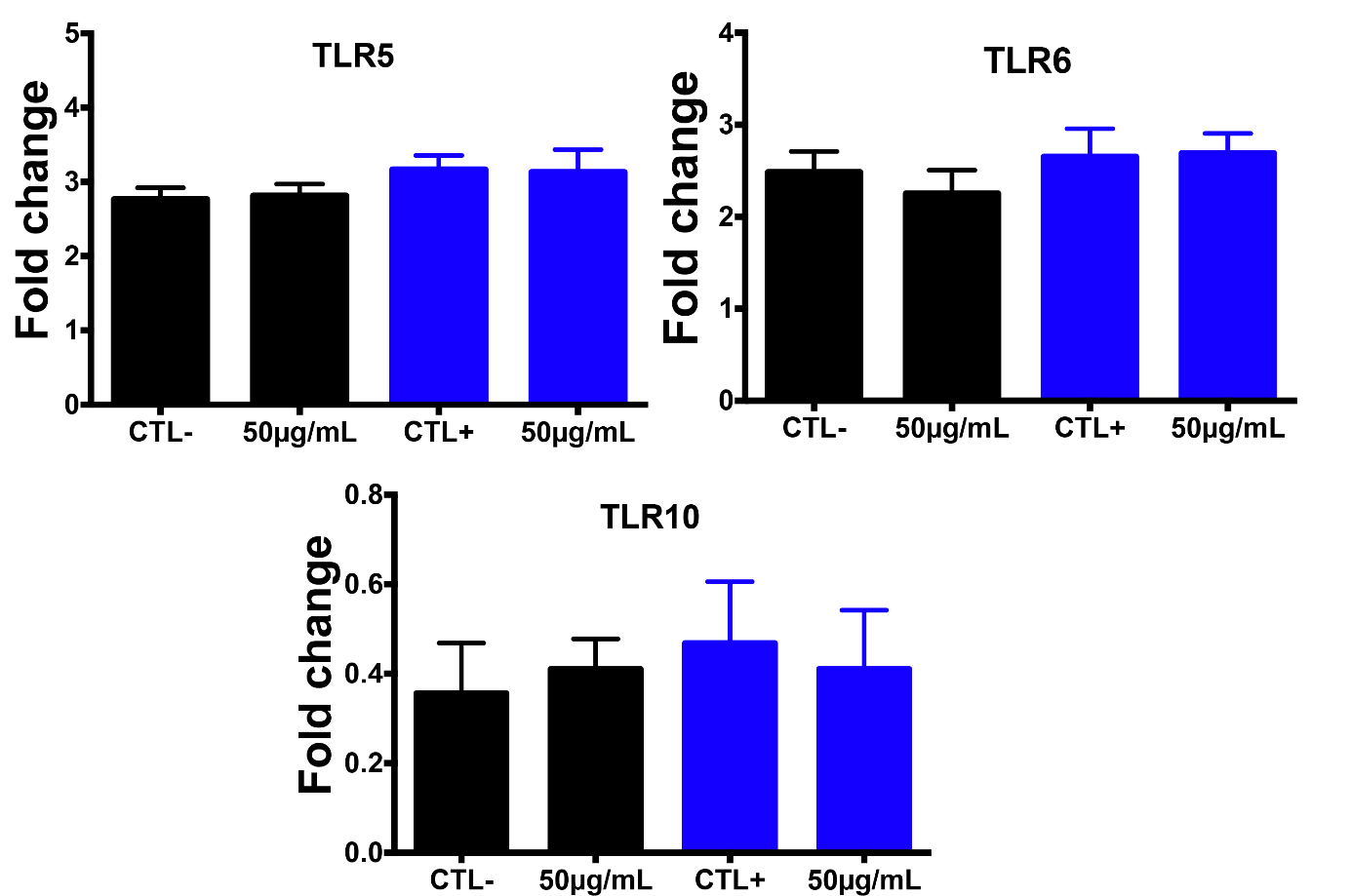
**

**Figure 2: Effect of chitin on TLR5, TLR6 and TLR10 expression in platelets.** Relative expression levels of TLR5, TLR6 and TLR10 mRNA, respectively, in platelets by real-time PCR. CTL- (control) corresponds to unstimulated platelets, CTL+ (control) represents platelets stimulated with thrombin (0.05 U/mL). 50 µg/mL corresponds to chitin concentration. Data are the mean ± SD of three independent experiments.

**
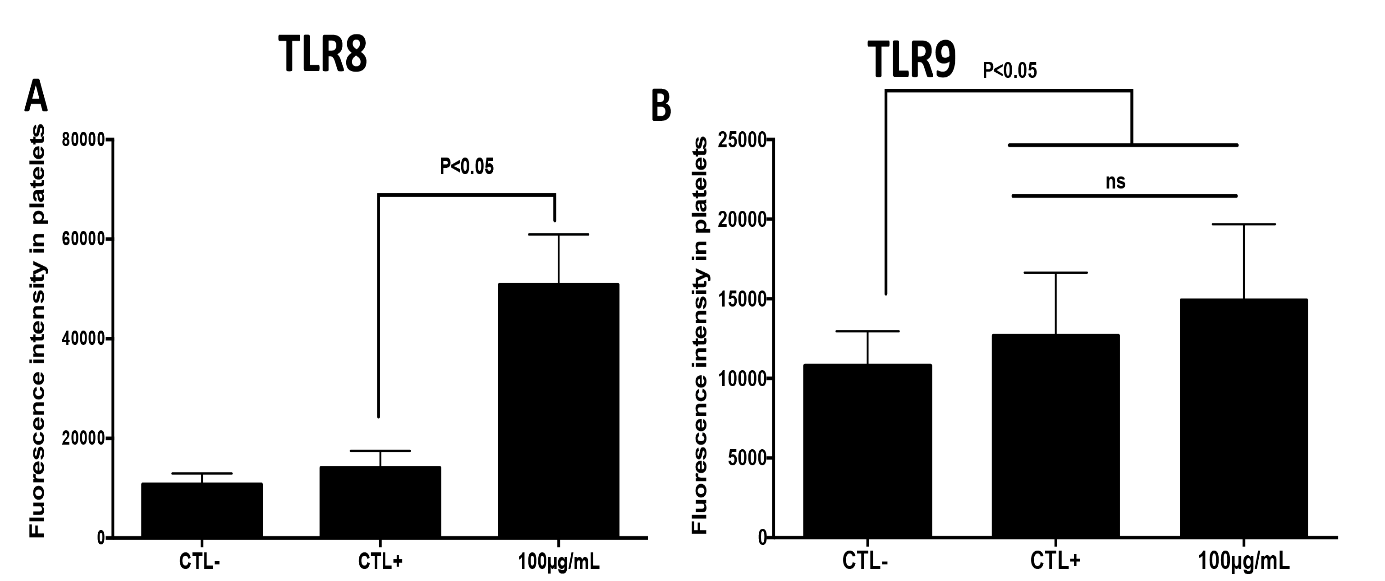
**

**Figure 3: Measurement of fluorescence intensity of TLR8 and TLR9 in platelets using ImageJ bundled with 64-bit Java 1.8.0-112.** Quantification of TLR8 and TLR9 fluorescence intensity was performed on 25 platelet cells from different images extracted from confocal microscopy using ImageJ bundled with 64-bit Java 1.8.0-112. CTL- (control) corresponds to unstimulated platelets, CTL+ (control) represents platelets stimulated with thrombin (0.05 U/mL). 100 µg/mL corresponds to chitin concentration.


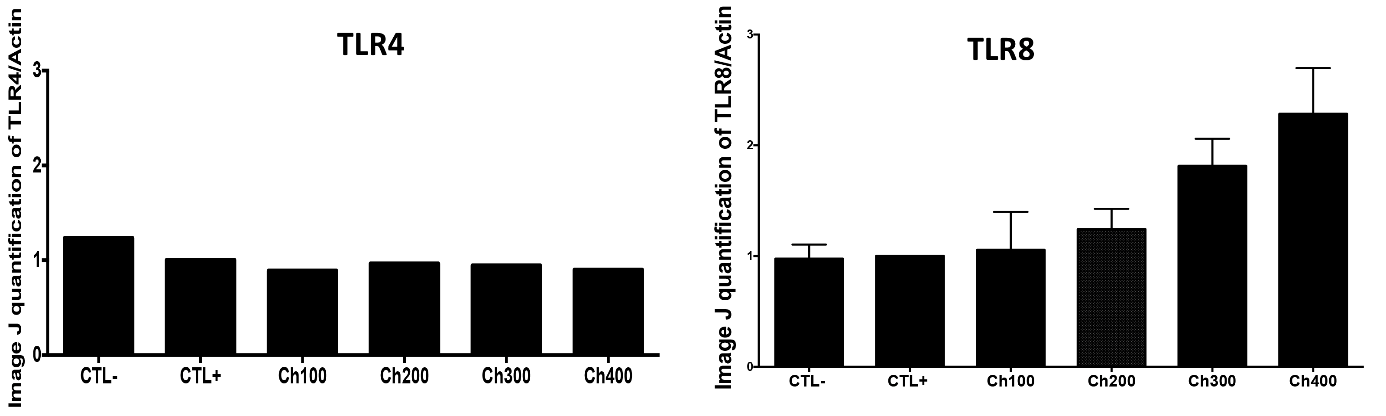


**Figure 4: Quantification of TLR4 and TLR8 Western Blot band intensities using ImageJ.** The intensity of each band was normalized to that of β-actin. CTL- (control) corresponds to unstimulated platelets, CTL+ (control) represents platelets stimulated with thrombin (0.05 U/mL). 100, 200, 300 and 400 µg/mL correspond to chitin concentration. Data are means ± SD of 3 independent experiments (n = 3). We observed an increase in TLR8 band intensity with an increase in chitin concentration.
